# Non-biased and efficient global amplification of a single-cell cDNA library

**DOI:** 10.1093/nar/gkt965

**Published:** 2013-10-18

**Authors:** Huan Huang, Mari Goto, Hiroyuki Tsunoda, Lizhou Sun, Kiyomi Taniguchi, Hiroko Matsunaga, Hideki Kambara

**Affiliations:** ^1^Biosystems Research Department, Hitachi Central Research Laboratory, 1-280 Higashi-koigakubo, Kokubunji-shi, Tokyo 185-8601, Japan and ^2^Maternity Department, First Affiliated Hospital of Nanjing Medical University, Guangzhou Road 300, Nanjing 210029, China

## Abstract

Analysis of single-cell gene expression promises a more precise understanding of molecular mechanisms of a living system. Most techniques only allow studies of the expressions for limited numbers of gene species. When amplification of cDNA was carried out for analysing more genes, amplification biases were frequently reported. A non-biased and efficient global-amplification method, which uses a single-cell cDNA library immobilized on beads, was developed for analysing entire gene expressions for single cells. Every step in this analysis from reverse transcription to cDNA amplification was optimized. By removing degrading excess primers, the bias due to the digestion of cDNA was prevented. Since the residual reagents, which affect the efficiency of each subsequent reaction, could be removed by washing beads, the conditions for uniform and maximized amplification of cDNAs were achieved. The differences in the amplification rates for randomly selected eight genes were within 1.5-folds, which could be negligible for most of the applications of single-cell analysis. The global amplification gives a large amount of amplified cDNA (>100 μg) from a single cell (2-pg mRNA), and that amount is enough for downstream analysis. The proposed global-amplification method was used to analyse transcript ratios of multiple cDNA targets (from several copies to several thousand copies) quantitatively.

## INTRODUCTION

Now that a large amount of sequencing data has been obtained by the Human Genome Project (HGP), the next big subject is to understand various biological phenomena from the viewpoint of system biology. As a single cell is the fundamental unit of a life system, single-cell analysis plays an important role in elucidating molecular mechanisms of a living system (1). However, due to technical limitations, most gene expression analyses are carried out with a large number of cells. They therefore give averaged data, which may mask important information. Significant cell–cell variations in stochastic gene expression (2) have been reported in studies on early embryonic development (3), neurosciences (4), stem cells (5), biophysical events in medicine (6) and disease (7); accordingly, technologies for single-cell analysis are urgently required.

Direct quantitative polymerase chain reaction (qPCR) analysis of a single-cell cDNA library without pre-amplification has been reported (8–10). Quantification of gene expression by qPCR is very accurate; however, the sensitivity of qPCR in regard to less-abundant transcripts is low, and the number of genes from a single cell that can be analysed at once is small. The sensitivity is low because a cDNA sample has to be divided into several fractions so that plural genes can be analysed. The authors therefore previously developed a method of combining qPCR and a bead-supported single-cell cDNA library (11). This method allows the repeated use of a whole-cDNA library for quantifying the expression of each gene. Accordingly, multiple genes can be analysed with a high enough sensitivity in regard to lowly expressed genes. However, the analysis of the entire mRNA is difficult in a short period of time because multiple genes have to be analysed in order. Although DNA chips (12) and next-generation DNA sequencers (e.g., the SOLiD system) (13) have been widely used for gene expression analysis of the entire mRNA in single cells, it requires global cDNA amplification (which frequently causes bias).

In this study, accordingly, all the processes included in global amplification of a cDNA library obtained from a single cell were evaluated, and an optimized uniform global-amplification method, based on bead-supported cDNA library preparation technology, was developed. A representation bias is negligible, meaning that the ratios of the cDNA copies for genes were unaltered after amplification. This method is applicable to sample preparation for gene expression analysis of the entire mRNA in single cells.

## MATERIALS AND METHODS

### Reagents

SuperScript® III, CellsDirect cDNA Synthesis Kit, Ribonuclease H (RNase H) and Lysis Enhancer were purchased from Invitrogen (Carlsbad, CA, USA). Terminal Deoxynucleotidyl Transferase (TdT), Recombinant, was purchased from New England Biolabs (Ipswich, MA). Exonuclease I was obtained from TaKaRa (Dailian, China). Streptavidin-coated beads (5 × 10^8^ beads, Φ = 1 µm, Dynabeads® MyOne™ Streptavidin C1) were purchased from Invitrogen (Oslo, Norway). Oligotex-dT30 Kit, ExTaq Hot Start Version and TaKaRa Premix ExTaq^TM^ were purchased from TaKaRa (Otsu, Shiga, Japan). RNase inhibitor, SUPERase-in RNase inhibitor, 10 × PCR Buffer II and 25-mM MgCl_2_ were obtained from Applied Biosystems (Foster City, CA, USA). Nonidet P40 (NP40) was obtained from Roche Diagnostics (Mannheim, Germany). Agencourt® AMPure® XP was purchased from Beckman Coulter, Inc. (Beverly, MA, USA). An RNeasy Mini Kit was obtained from Qiagen (MD, USA). T4 Gene 32 Protein was obtained from Wako Nippon Gene (Japan). A NdFeB magnet was obtained from Hitachi Ltd. (Japan). RT-PCR grade water was purchased from Ambion (Austin, TX, USA). High-sensitivity DNA reagents and chips were obtained from Agilent Technologies (Lithuania). All solutions were prepared in deionized and sterilized water. Other chemicals were of a commercially extra-pure grade.

### Primers sequences

Reverse-transcription (RT) primers and PCR primers were purchased from Sigma Genosys (Japan), and MGB probes were purchased from Applied Biosystems (America). The 5′-end of the RT primers (5′-ATA TGG ATC CGG CGC GCC GTC GAC TTT TTT TTT TTT TTT TTT TTT TTT-3′) was modified with dual biotin so that it could be immobilized on streptavidin-coated beads. Anchored UP2 primers (5′-ATA TCT CGA GGG CGC GCC GGA TCC TTT TTT TTT TTT TTT TTT TTT TTT VN-3′) were used for 2nd-strand cDNA synthesis. UP2 primers (5′-ATA TCT CGA GGG CGC GCC GGA TCC TTT TTT TTT TTT TTT TTT TTT TTT-3′) were used as a contrast. UP1 primers (5′-ATA TGG ATC CGG CGC GCC GTC GAC TTT TTT TTT TTT TTT TTT TTT TTT-3′) and anchored UP2 primers were applied to globally amplify cDNA during 20 cycles of the 1st PCR. Primers AUP1 (5′-amino-ATA TGG ATC CGG CGC GCC GTC GAC TTT TTT TTT TTT TTT TTT TTT TTT-3′) and AUP2 (5′-amino-ATA TCT CGA GGG CGC GCC GGA TCC TTT TTT TTT TTT TTT TTT TTT TTT-3′) were used for 15 cycles of the 2nd PCR. The sequences of qPCR primers and MGB probes for eight genes (EEF1G, B2M, TBP, SDHA, RPS18, RPL13A, GUSB and ATP5B) are listed in Table 1. The sequences of qPCR primers and MGB probes for four spike-in transcripts (‘spike 2’, ‘spike 3’, ‘spike 6’ and ‘spike 8’) are listed in Supplementary Table S1. The sequences of primers, which were used for preparing standard dsDNA templates immobilized on beads and free standard dsDNA templates are also listed in Table 1. All the oligonucleotides were HPLC purified.

**Table 1.** Primer sequences used for standard DNA preparation and qPCR

	Gene	Primer	Sequence (5′→3′)	Modification	Product size (bp)
Standard DNA	EEF1G	Forward	AGCTGCAATCTCATCACTGG		319
		Reverse	TGATGGCAAGAGATGTTCACTT	5′ dual biotin	
	B2M	Forward	CCGTGTGAACCATGTGACTT		264
		Reverse	CAACCTGCTCAGATACATCAAA	5′ dual biotin	
	TBP	Forward	GAGCTGTGATGTGAAGTTTCC		265
		Reverse	GGAGGCAAGGGTACATGAG	5′ dual biotin	
	SDHA	Forward	AGCACTGGAGGAAGCACAC		342
		Reverse	GAAGCAAGGGACAAAGGTAA	5′ dual biotin	
	RPS18	Forward	AGCCATGTCTCTAGTGATCCC		332
		Reverse	CCAGACCATTGGCTAGGAC	5′ dual biotin	
	RPL13A	Forward	CCTCAAGGTCGTGCGTCT		439
		Reverse	ACCTGCACAATTCTCCGAGT	5′ dual biotin	
	GUSB	Forward	AAGCCCATTATTCAGAGCGAGT		500
		Reverse	TTCCCTGCTAGAATAGATGACC	5′ dual biotin	
	ATP5B	Forward	TGCACGGAAAATACAGCGTT		364
		Reverse	GGGTGTACATTTTATTGGAAACCTT	5′ dual biotin	
qPCR	EEF1G	Forward	TTTCCGCTGAGTCCAGATT		149
		Reverse	CCCTGATTGAAGGCTTTG		
		Probe	TGGACTACGAGTCATACACA	5′ FAM/3′ NFQ, MGB	
	B2M	Forward	GCATCATGGAGGTTTGAAG		120
		Reverse	TATAACCCTACATTTTGTGCAT		
		Probe	CGCATTTGGATTGGATGA	5′ FAM/3′ NFQ, MGB	
	TBP	Forward	ACCCACCAACAATTTAGTAGTTAT		131
		Reverse	GCTCTGACTTTAGCACCTGTTA		
		Probe	AGCCAGAGT TATTTCCTGG	5′ FAM/3′ NFQ, MGB	
	SDHA	Forward	CACTGGGAAGGTCACTCTG		123
		Reverse	TTCTGTCATCACCACATCTTG		
		Probe	CCATTCGCTCCTACTGAT	5′ FAM/3′ NFQ, MGB	
	RPS18	Forward	ACTCACTGAGGATGAGGTGGA		137
		Reverse	CCAGACCATTGGCTAGGAC		
		Probe	TTGAACAGACAGAAGGATGTA	5′ FAM/3′ NFQ, MGB	
	RPL13A	Forward	TCATGAGGCTACGGAAACAG		125
		Reverse	CAACGCATGAGGAATTAACAGT		
		Probe	CCGAGAAGAACGTGGAGA	5′ FAM/3′ NFQ, MGB	
	GUSB	Forward	TGAACAGTCACCGACGAGAG		156
		Reverse	TCCAAACATTGTGACTTGGCTAC		
		Probe	CAGCGTTCCTTTTGCGAG	5′ FAM/3′ NFQ, MGB	
	ATP5B	Forward	CAGCAGATTTTGGCAGGTGAATA		129
		Reverse	GACAAAGACCCCTCACGATG		
		Probe	TGATAAGCTGGCTGAAGAG	5′ FAM/3′ NFQ, MGB	

### Cell culture and single-cell sampling

HCT 116 cells (CCL-247, ATCC) were cultured in a 25-cm^2^ flask containing advanced DMEM medium (Invitrogen) supplemented with 10% FBS (Invitrogen) under 5% CO_2_ at 37°C for 2 days. For cell dissociation, at first, cells were rinsed with PBS. After the cell-containing solution was kept at 37°C for 3 min with 0.25%-trypsin-EDTA (Invitrogen), a medium with 10% FBS was added to the solution. By centrifugation of the solution at 300 rpm for 3 min at 25°C, the supernatant was removed. Finally, 3 ml of PBS was added to re-suspend the cells. Before single cells were captured, the solution was diluted by PBS (37°C) to ∼0.5–2 cells/μl. After the diluted solution including the cells was put on a HydroCell 6-cm dish (CellSeed) kept on a thermo plate (CellSeed) at 37°C, a capillary tip (Φ = 190 μm; Drummond Scientific) was used to manually pick up a single cell in 0.5 μl of PBS under a microscope. The single cell was then transferred into a PCR tube containing cell-lysis solution with RT primer-supported beads on ice. All of the tips used in the cell culture and single-cell sampling were ART 200 g/1000 g self-sealing barrier tips (Molecular BioProducts). The processes from cell sampling to cell lysis were completed within 30 min.

### Preparation of standard mRNA samples

As for evaluating the proposed global-amplification method, standard mRNA samples were prepared by extracting mRNA from many cells since gene expressions in single cells change from cell to cell. Total RNA was extracted from HCT116 cells by using the RNeasy Mini Kit. Followed by DNase treatment of the extracted total RNA, mRNA was obtained by using the Oligotex-dT30 Kit twice. After phenol-chloroform extraction and the ethanol precipitation of mRNA, the concentration of purified mRNA was measured by UV. A series of mRNA samples with different concentrations (4 pg/µl, 40 pg/µl, 400 pg/µl and 4 ng/µl) was prepared by THT buffer (0.1% Tween-20, 10-mM Tris–HCl, pH 8.0) dilution. The amounts of mRNAs in 0.5 µl of the diluted samples corresponded to those for 1, 10, 100 and 1000 cells, respectively.

### Preparation of spike-in transcripts samples

Four kinds of PCR products (referred to as ‘spike 2’, ‘spike 3’, ‘spike 6’ and ‘spike 8’ hereafter) were amplified with a forward primer anchored to a T7 promoter sequence and a reverse primer anchored to an oligo(dT)_30_ sequence. The sequences of PCR primers and the sizes of the products are listed in Supplementary Table S1. After checking the electropherograms of the products with a bioanalyzer (Agilent 2100), the excess primers were removed from the products using a QIAquick PCR purification kit. After the ethanol precipitation of DNA, the concentrations of DNA were measured by UV absorption. RNA was synthesized by incubating 500 ng of each PCR product at 37°C for 1 h in a 10-μl reaction mixture containing 90 nmol of dATP, dCTP, dGTP and dUTP, 10 nmol of DTT, 1 μl of AmpliScribe T7-Flash Enzyme (Epicentre Biotechnologies) and 1 × AmpliScribe Reaction Buffer. The RNA samples were purified by DNase and protease-K treatments, after which phenol-chloroform extraction of the RNA samples was carried out twice. The residual dNTP in the purified RNA samples was then removed with an Oligotex-dT30 Kit, and the phenol-chloroform extraction of the samples was performed again. After ethanol precipitation of the samples, the pellets of the samples were re-suspended in 100 μl of RT-PCR grade water, and the RNA concentrations were measured by UV absorption.

### Preparation of RT primer-immobilized beads

Streptavidin-coated beads were suspended in 50 µl of binding and washing buffer (20 mM Tris–HCl (pH 8.0), 0.5 mM EDTA and 1 M NaCl) after being washed with 50 µl of the buffer three times. The dual-biotinated RT primers were diluted with the buffer and mixed to make a solution containing 4 × 10^10^ copies/μl of the primer molecules. The primers were immobilized on beads by adding 50 µl of the primer solution to the same volume of streptavidin-coated beads and mixing them at 750 rpm at room temperature for 1 h. The primer-immobilized beads were washed three times with 100 µl of binding and washing buffer and then washed three times with 100 µl of washing buffer [0.1% Tween 20, 10 mM Tris–HCl (pH 8.0)]. After the washing buffer was removed, the beads were suspended in 50 µl of washing buffer. Each bead had 4 × 10^3^ copies of RT primers on its surface. Before the primer-immobilized beads (10^7^ beads/μl) were used, they were washed thoroughly with equivalent amount of washing buffer to completely remove RT primers adsorbed on the surface of beads.

### Preparation of bead-supported cDNA libraries

A 4.05-µl cell-lysis solution of 0.9 × PCR Buffer II, 1.35-mM MgCl_2_, 0.45% NP40, 4.5-mM DTT, 0.18-U/µl SUPERase-in RNase inhibitor, 0.36-U/µl RNase inhibitor, 0.045-mM dNTP and a set of spike-in transcripts (10 copies of spike-2 RNA, 50 copies of spike-3 RNA, 200 copies of spike-6 RNA and 1000 copies of spike-8 RNA) were added to a 0.2-ml tube containing 10^7^ RT primer-immobilized beads. Totally, ∼4 × 10^10^ copies of RT primers with dual biotin at the 5′-end were immobilized on surfaces of 10^7^ beads. Then, mRNA or a single cell in 0.5 μl of PBS was added to the solution and mixed gently. After the solution was heated at 70°C for 1.5 min and then gradually cooled down to 4°C, 0.45 μl of RT mixture (0.40-U/µl RNase Inhibitor, 0.07-µg/µl T4 Gene 32 Protein and 13.2-U/µl SuperScript III) was added to the cooled solution to be mixed. RT was carried out by shaking the tube at 750 rpm at 50°C for 30 min in a microincubator (Taitec, M-36). The solution was then heated at 70°C for 10 min to deactivate enzymes.

### Preparation of amplified cDNA libraries

After RT, the supernatant was removed by capturing the beads on the tube surface with an NdFeB magnet. The beads were washed twice with THT buffer and re-suspended in 12 μl of poly(A)-tailing solution [0.6-μl 10 × PCR Buffer II, 0.75-mM MgCl_2_, 1.5-mM dATP, 0.6-U RNase H, 4.5-U TdT, 0.05% Tween-20 and 5-mM Tris–HCl (pH 8.0)]. The sample was incubated at 37°C for 15 min to promote a poly(A)-tailing reaction. After the tailing enzyme was inactivated (70°C for 10 min), the sample (12 μl) was divided into four equal fractions. To each fraction (containing 3 μl of the sample), 19 μl of PCR mixture I (1.9-μl 10 × EX *Taq* Buffer, 0.25-mM dNTP, 0.3-μM anchored UP2 Primer and 0.95-U TaKaRa ExTaq^TM^ HS) was added and the 2nd-strand cDNA synthesis was performed. Each fraction was kept in a thermal cycler (Applied Biosystems) at 95°C for 3 min, at 44°C for 2 min and at 72°C for 6 min. Global amplification of a cDNA library was then carried out after adding 19 μl of PCR mixture II (1.9-μl 10 × EX *Taq* Buffer, 0.25-mM dNTP, 2.2-μM UP1 and 0.95-U TaKaRa ExTaq^TM^ HS) to the fraction. The product was held at 95°C for 3 min, and then subjected to 20 thermal cycles of 95°C for 30 s, 67°C for 1 min and 72°C for 6 min (+6 s each cycle). After the beads were removed from the reaction tubes, the PCR products in the four tubes were combined and purified by Agencourt® AMPure® XP twice to remove primer-dimers. For the purification, the optimal volume ratio of PCR products to AMPure-beads solution is 1–0.6. Another 15 cycles of PCR was then performed on the four fractions, each containing 1 μl of the 50 -μl purified PCR products together with 49 μl of PCR mixture III (5-μl 10 × EX *Taq* Buffer, 0.25-mM dNTP, 1-μM AUP1, 1-μM AUP2 and 2.5-U TaKaRa ExTaq^TM^ HS). Each fraction was held in a tube at 95°C for 3 min and subjected to 15 thermal cycles of 95°C for 30 s, 67°C for 1 min and 72°C for 6 min (+6 s each cycle). The amplified cDNA library was obtained by collecting the products from the four tubes.

### Quantitative analysis of genes in single cells as well as pooled cell samples

Expression levels of eight genes (EEF1G, B2M, TBP, SDHA, RPS18, RPL13A, GUSB and ATP5B) and four spike-in transcripts (‘spike 2’, ‘spike 3’, ‘spike 6’ and ‘spike 8’) were measured with a qPCR system (Applied Biosystems, ABI PRISM 7900, version 2.1). Standard dsDNA templates immobilized on beads were prepared as the references for obtaining standard curves for the eight genes (11). A reaction mixture for the qPCR analysis contained 1 × Premix Ex *Taq*, 1 μM of each qPCR primer pair, 0.25-μM TBP MGB fluorogenic probes, 0.19% PMB 30 and 4 μl of a cDNA library sample (or 1 μl of a standard dsDNA sample) immobilized on 10^7^ beads. For the analysis of free cDNA samples, free standard dsDNA templates were prepared by diluting purified PCR products at a series of concentrations. The reaction mixture for the qPCR analysis of a free sample contained 1 × Premix Ex *Taq*, 1 μM of each qPCR primer pair, 0.25-μM TBP MGB fluorogenic probes, 1.8-mM Tris–HCl, 0.018% Tween 20 and 1 μl of a free cDNA library sample or a free standard dsDNA sample. The expression levels of the eight genes in the samples were quantitatively analysed by detecting fluorescence signals during thermal cycles (95°C for 30 s, followed by 40 cycles of 95°C for 5 s and 60°C for 30 s). The copy numbers of cDNA molecules in the samples were estimated from the standard curves obtained with the standard templates.

## RESULTS

### Flow of sample preparation for preparing an amplified cDNA library from single cell

**Figure 1.**
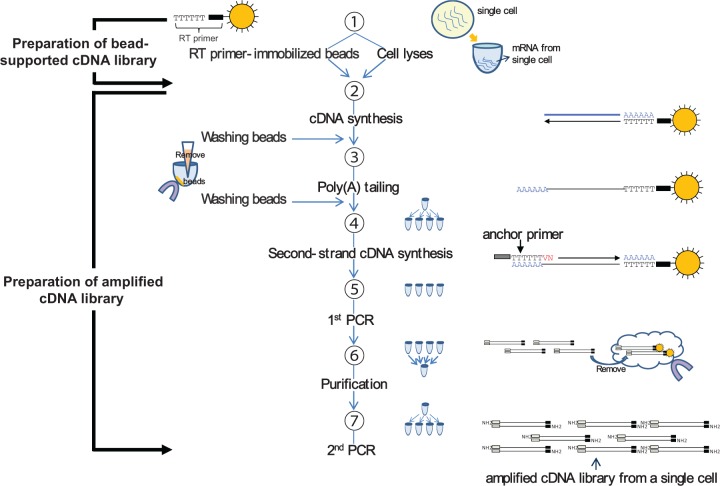
Sample-preparation flow for non-biased global amplification of a cDNA library from single cell.

An outline of the flow of the sample preparation is shown in Figure 1. It includes processes for preparing a single-cell cDNA library on beads and globally amplifying all cDNAs in the library. The protocol includes the following steps. Firstly, mRNA is released from a cell by lyses. After removing genomic-DNA by digesting with DNase, mRNAs are hybridized with RT primers immobilized on beads. RT is carried out to produce bead-supported cDNAs. Unlike free cDNAs (which are commonly used in RT), bead-supported cDNAs can easily be washed to remove the RT solution while being held with a magnet. Although the digestion of RT primers with exonuclease I is frequently used to remove the residual primers, the digestion process is skipped here. The poly(A)-tailing reaction is carried out directly after the washing process. To keep the concentrations of 4 nt equal for the later PCR reaction, the beads are washed again to remove the residual dATPs. After the washing, one sample is divided into four fractions so that the 2nd-strand cDNA synthesis is carried out in parallel with hybridizing anchor primers on the synthesized poly(A) tails. Common primers and PCR reagents are then supplied before the 1st PCR cycle. After the 1st PCR amplification, the products in the four tubes are mixed together and the beads are then removed. As primer-dimers produced in the 1st PCR interrupt the amplification of target cDNA in the 2nd PCR, they are removed before the 2nd PCR of 15 cycles in the 4 tubes to avoid unbiased amplification. The four products of the 2nd PCR are mixed to prepare the final sample for downstream analysis.

### Bias produced in the preparation processes of single-cell cDNA library

The original global-amplification method (13) includes steps for cDNA synthesis, exonuclease-I treatment, poly(A) tailing, 2nd-strand cDNA synthesis and PCR. Since there are many possible sources producing a bias, each step was evaluated carefully here in terms of the bias it produced.

**Figure 2.**
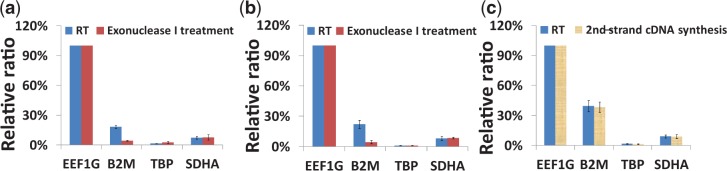
Relative abundances of transcripts in cDNA libraries obtained by qPCR. The copy number of cDNA for gene EEF1G was set to 100% as a reference for calculating relative ratios of cDNA for the other three genes (B2M, TBP and SDHA). The error bars are independent amplification replicates. (**a**) For a bead-supported cDNA library treated with exonulease I, the ratios were obtained with samples after RT (blue column) and after exonulease-I treatment (red column). (**b**) For a cDNA library free in an RT solution treated with exonulease I, the ratios were obtained with samples after RT (blue column) and after exonulease-I treatment (red column). (**c**) For a bead-supported cDNA library treated without exonulease I, the ratios were obtained from samples after RT (blue column) and after 2nd-strand cDNA synthesis (yellow column).

**Figure 3.**
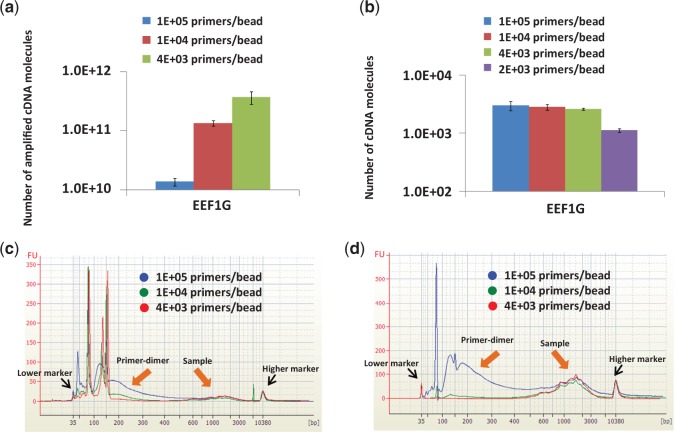
Change of products produced with 10^7^ beads immobilizing RT primers with various densities. Starting material in all cases was 2 pg of mRNA. The error bars are independent amplification replicates. (**a**) Number of amplified cDNA molecules (EEF1G gene) in cDNA libraries after 2nd PCR. (**b**) Number of cDNA molecules (EEF1G gene) produced on beads in RT. (**c**) Electrophoregrams of crude 1st PCR products. (**d**) Electrophoregrams after purifying the crude 1st PCR products showed in the electrophoregrams of graph ‘c’.

At first, a bias produced in degrading RT primers with exonuclease I was evaluated. Exonuclease I is frequently used to digest unincorporated single-stranded primers in a reaction mixture containing double-stranded extension products (12–14). It is also used in a commercialized reagent kit (CellAmp® Whole Transcriptome Amplification Kit) to reduce the reaction by-products. When the exonuclease is not used to digest the excess primers, they are tailed with poly(A), which competes with the cDNA targets in the later PCR processes, resulting in low amplification efficiency. However, it was found that unstable cDNA/RNA duplexes were also degraded in the digestion step. The degradation changed the relative abundance of transcripts (e.g., B2M gene in Figure 2a and b; RPL13A and ATP5B in Supplementary Figure S1). These effects occurred in the case of a bead-supported cDNA library (Figure 2a; Supplementary Figure S1) and in the case of a cDNA library free in an RT solution (Figure 2b). Removing the degradation process prevented the digestion of cDNA; thus, a bias due to cDNA digestion was avoided (Figure 2c). In terms of quantitative analysis of gene expression in single cells, the degradation bias should be avoided. When the residual RT primers were not digested, they were poly-A tailed which produced so many primer-dimers and interrupted the latter cDNA amplification process. Instead of using exonuclease, the amount of bead-supported RT primers was reduced as much as possible. When the RT primer amount was decreased from 10^5^ to 4 × 10^3^ copies/bead in a reaction volume of 5 µl with 10^7^ beads, the total amplification efficiency by the two-step PCR increased by ∼30 times (Figure 3a). Moreover, RT efficiency did not decrease significantly under the RT condition mentioned above (Figure 3b). The high amplification efficiency was due to the significant reduction in the production rate of primer-dimers in the 1st PCR (Figure 3c) coupled with easy purification of the products by using the purification kit of Agencourt® AMPure® XP (Figure 3d). It is important to keep the amount of beads as 10^7^ while the number of probes on a bead was decreased. Even when the total number of probes (4 × 10^10^ copies) was the same, the RT efficiency was reduced by decreasing the amount of beads to 10^6^ (data not shown). This primer-beads ratio (4 × 10^3^ copies/bead with 10^7^ beads for an RT reaction) works well for <200-pg mRNA (Supplementary Figure S2). This amount of primers seems good enough for carrying out RT reaction efficiently with the small amount of mRNA. Low RT efficiency which was found by analysing a large amount of mRNA (2 ng mRNA) might due to the insufficient amount of primers for a large amount of mRNA. As for analysing a small amount of mRNA (i.e., 0.5 pg mRNA or less) in single cells, this small amount of primers and this low primer-beads ratio are suitable because they are enough for capturing a very small amount of mRNA.

**Figure 4.**
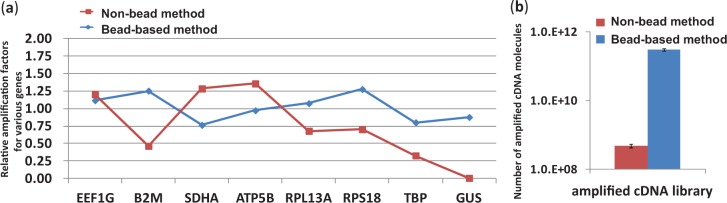
Comparison of amplification bias and amplification efficiency in the case of the bead-based method and the none-bead method. In all cases, starting material was 2 pg of standard mRNA. (**a**) Relative amplification biases for cDNA of eight genes. The amplification factors for the eight cDNAs were obtained by averaging the results of five trials. The geometric means were calculated to obtain the amplification biases (amplification bias: the ratio of an amplification factor for a gene to the geometric means). (**b**) Number of cDNA molecules (EEF1G gene) after amplification by the bead and non-bead methods. The error bars are independent amplification replicates.

Besides the bias produced during the degradation of RT primers, reactions in other steps may cause biases. To reduce the possible bias originated in stochastic errors, one sample was divided into four fractions for carrying out the same reactions of poly(A) tailing, 2nd-strand cDNA synthesis and PCR in parallel (12). After the reactions, the products in four tubes were got together for an analysis. Enzymatic reactions are frequently affected by the coexisting components in a reaction solution. The influence of residual reagents on subsequent reactions should therefore be eliminated. In the proposed method, it was eliminated completely by washing the beads. Whether a series of reactions were carried out homogeneously or not depends on uniform dispersion of beads in a solution. To avoid the risk of non-uniform dispersion of beads, the tubes were shaken with a microincubator during RT and poly(A)-tailing steps. Moreover, the beads were well dispersed in a reaction solution before the start of 2nd-strand cDNA synthesis and PCR. To minimize PCR bias, a two-step PCR with a rather small number of cycles and with a purification step between the two PCR was used instead of one-step PCR with more cycles. This set-up was effective to minimize the number of amplification cycles and to obtain a high amplification efficiency. Actually, the amplification efficiency for lowly expressed genes was increased. For example, the global-amplification method before optimization (marked ‘Non-bead method’ (13) in Figure 4a), failed to detect a lowly expressed gene, such as GUSB (<10 copies of mRNA expressed in one cell), and the bias among the detected genes was large (Figure 4a, red line). The proposed bead method (marked with ‘Bead-based method’ in Figure 4a) showed a low amplification bias of <1.5-folds (Figure 4a, blue line). All of the eight genes (including the lowly expressed gene such as GUSB) could be easily and accurately detected, although the used mRNA amount was as small as that in one cell. The good replications (∼30 times) by analysing genes expressed at low levels indicate that the detection limit of the bead-based method is as low as 5–10 copies per cell. Besides the low bias in amplification, cDNA could be amplified highly efficiently (Figure 4b). Comparing the qPCR results for the original cDNA molecules immobilized on beads and those for the amplified cDNA molecules in 2nd PCR products indicates that the total amplification rate averaged over four genes (EEF1G, B2M, TBP and SDHA) is more than 10^8^ times. It means that ∼200 μg of global cDNA library [2 pg × 10^8 ^= 200 μg could be obtained from single cells (2-pg mRNA)] can be used for downstream analysis. This amount of global cDNA library is much bigger than that obtained with the non-bead method (a nanogram amount). The absolute copy numbers of transcripts in single-cell level RNA were estimated by calculating the numbers of transcript molecules in pooled cell samples, which were confirmed by qPCR. Four kinds of spike-in transcripts (named ‘spike 2’, ‘spike 3’, ‘spike 6’ and ‘spike 8’) were used as the experimental controls to be analysed together with transcriptomes from single cells. The qPCR for the amplified spike-in transcripts show similar results (the negligible bias and the highly efficient amplification) with those obtained from single cells (Supplementary Figure S3).

### Effect of RT reagents on subsequent reactions

**Figure 5.**
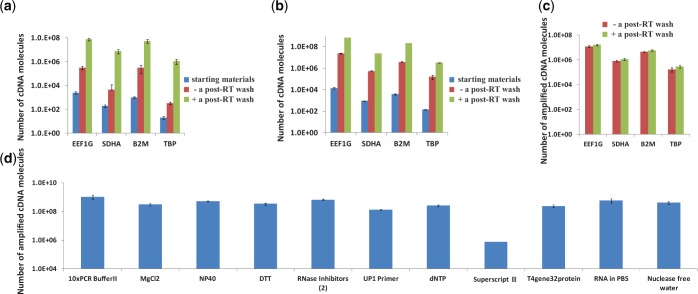
Affect of residual RT reagents on subsequent reactions. The number of cDNA molecules was evaluated by qPCR. Error bars are independent amplification replicates. (**a**) The residual RT reagents in a poly(A)-tailing reaction mixture containing bead-supported cDNA made biases large and amplification factors small (red column). The difficulty was overcome by washing beads (green column). (**b**) The addition of RT reagents to purified model cDNAs free in a poly(A)-tailing reaction mixture made biases large and amplification factors small as well. After purifying model cDNA with a purification kit, RT reagents were added to cDNA (red column) or not added (green column). (**c**) RT reagents did not affect the 2nd-strand cDNA synthesis and PCR. (**d**) Amplification efficiency was strongly affected by the presence of SuperScript III. The 11 components in an RT reagent kit were, respectively, added to 11 purified RT samples before poly(A) tailing. The 1st PCR products were evaluated by qPCR. Although the original amounts of samples were the same, the product produced by adding SuperScript III was small.

As enzymatic reactions can be affected by coexisting components in a solution, the influence of residual reagents on the later reactions should be investigated. Accordingly, the addition of RT reagents to a poly(A)-tailing reaction mixture containing bead-supported cDNA (Figure 5a) or cDNA free in the solution (Figure 5b) was investigated. It was found that this addition decreased the global-amplification efficiency several dozen times, namely, from 1.8 times/cycle (Figure 5a and b, green columns) to <1.5 times/cycle (Figure 5a and b, red columns). The amplification rates per cycle were calculated on the basis of the *N*th root of amplification rate averaged over four genes (EEF1G, B2M, TBP and SDHA). *N* is 18, namely, the number of cycles in PCR. However, the addition of RT and poly(A)-tailing reaction reagents to 2nd-strand cDNA synthesis and PCR reaction mixtures did not affect the synthesis and amplification efficiencies (Figure 5c). This result indicates that RT reagents inhibit a poly(A)-tailing reaction and should therefore be removed before the reaction.

It is rather difficult to remove RT reagents from a solution. Even if the purification of cDNA by a column is the best way, it is very difficult to collect cDNA, especially cDNA expressed at a low level, efficiently. The recovery rate of cDNA after the purification is frequently <30%. Besides, the reaction volume increases after the purification. These issues can be overcome by the use of a cDNA library on beads together with washing. To investigate the enzymatic inhibition of subsequent reactions, the effect of various RT reagents on these reactions was investigated. According to Figure 5d, only SuperScript III (RT enzyme) inhibits the subsequent enzymatic reactions significantly. However, SuperScript III is the most efficient RT enzyme suitable for preparation of a bead-supported cDNA library from a single cell (11). Accordingly, SuperScript III was used, and after the reaction, it was removed by washing.

### Elimination of primer-dimers

**Figure 6.**
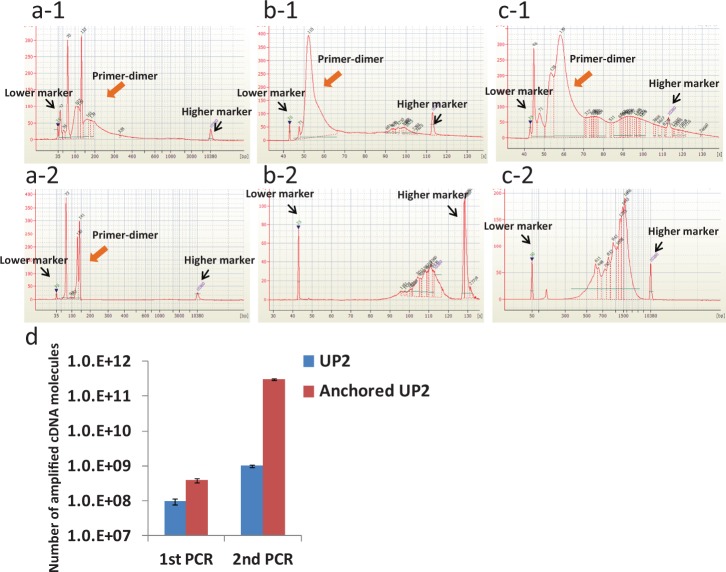
Reduction of primer-dimer production by introducing VN sequence to UP2 primers. Although the production of primer-dimers by PCR is a big issue in terms of whole-cDNA amplification, it is greatly reduced by the use of UP2 primers with poly(T) plus VN sequences at 3′ termini. Electrophoregrams of PCR products obtained with two different primers (denoted as 1 and 2 in the figures) for the 2nd-strand synthesis are shown in figures (**a1–c2**). In the figures, ‘a’, ‘b’ and ‘c’ stand for crude 1st PCR products, purified 1st PCR products and 2nd PCR products, respectively. The primers used in the cases of Figures a1, b1 and c1 were UP2 primer having poly(T) sequence at 3′ termini. The primers used in the cases of Figures a2, b2 and c2 were anchored UP2 primers having poly(T) plus VN sequences at the 3′ termini. (**d**) The number of amplified cDNA molecules (EEF1G gene) after the 1st PCR, as well as the 2nd PCR, obtained with the two kinds of primers for 2nd-strand synthesis.

The formation of primer-dimers is problematic in terms of obtaining both efficient global amplification and downstream analysis. In the two-step PCR, primer-dimers produced in the 1st PCR are removed with purification kits efficiently as long as the lengths of primer-dimers are short (i.e., <200 bp) and the population of their sizes is localized. However, the population frequently becomes widely spread when the 3′-end of the UP2 primers used in the 2nd-cDNA strand synthesis are poly(T) (13,15). The poly(T) primers hybridize with tailed poly(A) sequences at various positions and then extend their lengths to make a broad size profile (Figure 6a-1), which results in a low purification efficiency for primer-dimers (Figure 6b-1) and a high amplification efficiency for dimers (Figure 6c-1). These problems were overcome by using anchored UP2 primers with poly(T) plus *VN* (where *V* is dA, dC or dG and *N* is dA, dC, dG or dT) so that the 3′-end of the primers can hybridize only at the 5′-end of the poly(A) tail of cDNAs. The use of the anchored UP2 primers made the size profile of the primer-dimers narrow (Figure 6a-2) enough for an efficient purification (Figure 6b-2) to increase the amplification efficiency in the 2nd PCR (Figure 6c-2) by several dozen times (Figure 6d).

### Application of the bead-based method for single-cell analysis

**Figure 7.**
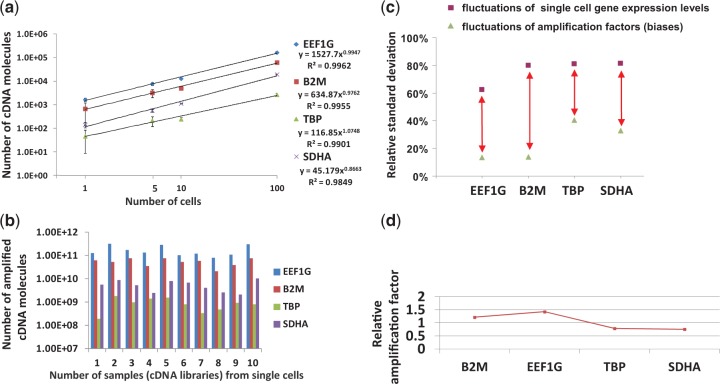
Single-cell analysis with the proposed bead-based method. (**a**) Linear relation between gene expression levels and number of cells (average number of mRNA molecules) in pools. The error bars are independent amplification replicates (single cell: mean ± SD, *n* = 10; 5–100 cells: mean ± SD, *n* = 3). (**b**) Cell-to-cell variations in gene expression among single cells. The number of amplified cDNA molecules in single cells was measured after the 2nd PCR. (**c**) Relative standard deviations of the amplification factor for each of four genes (EEF1G, B2M, TBP and SDHA) (*n* = 15) and gene expression levels in single cells (*n* = 30). The fluctuation of biases for every trial is smaller than the fluctuation of gene expression levels in single cells. (**d**) Relative amplification bias for four genes. Gene expressions of 4 genes in 30 unamplified bead-supported single-cell cDNA libraries and those in 10 amplified single-cell cDNA libraries were quantitatively analysed by qPCR. The amplification factors for the four genes were obtained by averaging the ratios of the cDNA copies after the amplification (*n* = 10) to those before the amplification (*n* = 30). The geometric means were calculated to obtain the amplification bias (amplification bias: the ratio of an amplification factor to the geometric mean).

Since gene expressions in single cells change from cell to cell, it is difficult to evaluate the reproducibility of the method with real single-cell samples. Accordingly, a series of pooled cell samples (5, 10 and 100 cells), together with single-cell samples, were prepared. The average number of cDNA molecules is in proportion to the number of cells (Figure 7a), indicating that the number of cDNA molecules for a single-cell sample was reasonable. Gene expressions for 4 genes in 40 single-cell cDNA libraries were quantitatively analysed. The transcript ratios in 30 unamplified bead-supported single-cell cDNA libraries were compared with those in 10 amplified single-cell cDNA libraries. The observed biases for the four genes were within 1.5-folds (Figure 7d). This result is consistent with the results obtained with pooled cell cDNA libraries (within 1.5-folds, Figure 4a). Those results indicate that the bead-based method is applicable not only to model samples but also to real single-cell samples and that the biases among gene species are small. The low bias was also evaluated by analysing another 12 genes in Supplementary Figure S4. The amplification biases for the 12 genes were also within 1.5-folds. Moreover, relative standard deviation of the amplification factors are smaller than that of gene expression levels in single cells, indicating that the method can successfully be used to analyse changes of gene expression levels in single cells and the fluctuation of biases is small (Figure 7c). Since the use of beads affords washing, every process can be carried out under optimum conditions, and almost every one of a number of independent amplifications (∼100 times) could successfully analyse the single-cell level RNAs.

The transcript ratios of 4 genes for the 10 amplified single-cell cDNA libraries are shown in Figure 7b. The differences in the transcript ratios (a maximum of 6-folds) are much larger than the amplification bias of transcripts (a maximum of 1.5-folds, mentioned above). Moreover, in Figure 7a, the coefficient of variation in the number of cDNA molecules for the single cells was larger than that for 5 cells and much larger than those for 10 and 100 cells. These results indicate cell–cell variations in stochastic gene expression. The proposed method makes it possible to discover the heterogeneity (several sub-groups) in isogenic stem cells and the change in the number of cells in individual groups during induction. This application of the proposed method will thus be helpful to elucidate the differentiation mechanisms of stem cells.

## DISCUSSION

The ability to analyse genome-wide transcriptomes has tremendous potential to unlock a wealth of biological information. However, it has been technically challenging to generate expression profiles from single cells, especially bias, such as PCR-introduced bias, sequence-dependent bias and degradation-induced bias, which is always produced by technical limitations. Accordingly, a non-biased global cDNA library amplification method for gene expression analysis of entire mRNAs in single cells was successfully developed. The main technical novelty of this work is the combination of bead-supported cDNA library preparation and unbiased amplification of cDNA from single cells. Although the process of removing excess primers with exonuclease I, which can improve the amplification efficiency, was neglected due to its negative effect on uniform global amplification, primer-dimers were still suppressed and a high amplification efficiency was achieved by using beads for supporting cDNA, changing to an anchored primer, minimizing RT primers on beads and removing SuperScript III. A high-quality cDNA library prepared in this work is preferable for downstream analysis. The proposed method is suitable for accurate analysis of significant cell–cell variations in stochastic gene expression and discovery of many new transcripts in single cells.

Other popular approaches, such as SMART-seq (16) and CEL-seq (17), avoid exonuclease-bias because these approaches do not require primer degradation with exonuclease I and poly(A) tailing. However, they have their own limitations, namely, bias and relatively low amplification efficiency. Only a few nanograms of cDNA can be obtained by amplification of full-length cDNAs from 10 pg of total RNA or from single cells in the case of SMART-seq (16). This amount is much less than that (>100 μg of amplified cDNA) obtained from a single cell (2-pg mRNA) in the case of the bead-based method. Moreover, incomplete cDNAs are discarded. The loss of the information on the non-full-length cDNAs might result in low sensitivity for low-level transcripts and possible bias. In the case of CEL-seq, as the linear amplification mode of *in vitro* transcription (IVT) was used, the sensitivity is low in regard to analysis of entire transcripts, especially low-level transcripts. In comparison with micrograms of cDNA (>100 μg) obtained with the proposed global-amplification method using an exponential amplification, only nanograms of cDNA can be produced from 2-pg mRNA by CEL-seq. The amount of cDNAs obtained with the proposed global-amplification method (bead-based method) is sufficient for DNA downstream analysis. The proposed bead-based method has a high enough sensitivity for analysing almost all transcriptomes from a single cell, especially for low-abundance transcripts (<10 copies per cell). The high sensitivity is particularly important for early embryo studies because some of the key transcriptomes are expressed at very low levels. In the proposed work, although the limitations of making a cDNA library from 3′ termini of mRNA to produce cDNA with partial lengths frequently still exist, the future improvement as to RT condition might be possible to overcome these limitations.

The cDNA preparation/amplification bias was evaluated with qPCR for eight genes in this work because qPCR has its own unique advantages of greater sensitivity, accuracy and specificity for the transcript profiles compared to conventional microarrays and sequencing (18). The reasons that qPCR, not microarray or sequencing, was applied in the proposed bead-based method are as 4-folds. First, it is difficult to accurately analyse a small amount of mRNA by sequencing or with a DNA chip, although qPCR can do it easily. It was necessary to compare the quantitative analysis results obtained with a small amount of sample and those obtained with its amplified sample. Second, qPCR is considered to be the most accurate quantitative analysis method that can be applied to a wide range of copy numbers (from several copies to several thousand copies). Gene species expressed at various levels were selected as the representatives. Third, the use of sequencing with next-generation DNA sequencers produces additional bias due to amplified cDNA shearing (fragments of 80–130 bp) and adaptor ligation followed by fragment amplification. Mapping of the read fragments onto genomes to obtain expression levels also causes different biases in view of different amounts of samples. Fourth, because many fragments for sequencing will have chimeric sequences containing the anchored primers, poly(A), etc. that will not align, there remains a cumbersome bioinformatic challenge to aligning the reads of the fragments to a genome reference. The necessity of pruning the data and discarding many fragments makes it unclear what depth of sequencing would be required to obtain an accurate gene expression profile (19).

The major technical advantages of the proposed bead-based method are summarized as follows: (i) optimum conditions for reactions can be easily attained by washing the magnetic beads immobilizing a cDNA library; (ii) ratios of cDNA molecules in single cells can be maintained during amplification; (iii) highly efficient amplification of all cDNAs (high enough for downstream analysis); (iv) process reproducibility is achieved because most ambiguities due to coexisting components are removed; and (v) a reusable single-cell cDNA library immobilized on beads is produced by collecting the cDNA library on beads after 1st PCR with a magnet. The recovered cDNA library can be used for the further gene expression analysis anytime in the future if necessary.

Since it is very easy to handle a single-cell cDNA library on beads by using a magnet, we believe that high-efficiency and high-fidelity reactions can be achieved by using bead for supporting the single-cell cDNA library and by washing beads to attain the optimum reaction conditions for every step in the popular other approaches for gene expression analysis. Moreover, the reusability of a bead-supported cDNA library can also be attained in the popular other approaches. As every step in the proposed bead-based method is easy for anyone to carry out, this non-biased and efficient global-amplification method based on a bead-supported cDNA library is promising for studies on cell–cell variations, fundamental biological researches and applications in various biological contexts (including diagnosis).

## SUPPLEMENTARY DATA

Supplementary Data are available at NAR Online.

## FUNDING

Innovative Cell Biology by Innovative Technology (Cell Innovation Program) from Ministry of Education, Culture, Sports, Science and Technology (MEXT, in part); National Natural Science Foundation (NSFC) [21305069]. Funding for open access charge: MEXT.

*Conflict of interest statement*. None declared.

## Supplementary Material

Supplementary Data
