# Supplementary material for: Non-biased and efficient global amplification of a single-cell cDNA library
Source: Nucleic Acids Res. 2013 Oct 18;42(2):e12. doi: 10.1093/nar/gkt965 (PMC3902946; doi:10.1093/nar/gkt965)
Supplement: Supplementary Data [file supp_42_2_e12__index.html]

Non-biased and efficient global amplification of a single-cell cDNA library — Non-biased and efficient global amplification of a single-cell cDNA library — Supplementary Data 

# Non-biased and efficient global amplification of a single-cell cDNA library

## Supplementary Data

files

**Files in this Data Supplement:**

- Supplementary Data - doc file
