# Supplementary material for: Non-biased and efficient global amplification of a single-cell cDNA library
Source: Nucleic Acids Res. 2013 Oct 18;42(2):e12. doi: 10.1093/nar/gkt965 (PMC3902946; doi:10.1093/nar/gkt965)
Supplement: Supplementary Data [file supp_gkt965_nar-02214-met-g-2013-File003.doc]

Supplementary Table 1. Primer sequences used for spike-in transcript preparation and qPCR.

|  | Gene | Primer | Sequence (5’→3’) | Modification | Product size (bp) |
| --- | --- | --- | --- | --- | --- |
| Spike-in transcripts | Spike 2 | Forward | TAATACGACTCACTATAGGGCTTCGATTCTGTTTTGCTAC |  | 750 |
|  |  | Reverse | TTTTTTTTTTTTTTTTTTTTTTTTTTTTTTCTATACAAAACCGTGCGCGG |  |  |
|  | Spike 3 | Forward | TAATACGACTCACTATAGGGCTTCGGCAACATTAACTGG |  | 1000 |
|  |  | Reverse | TTTTTTTTTTTTTTTTTTTTTTTTTTTTTTACGCGTTATAGGCATTCTCG |  |  |
|  | Spike 6 | Forward | TAATACGACTCACTATAGGGGGGCTGGAGATCATCCTAC |  | 1250 |
|  |  | Reverse | TTTTTTTTTTTTTTTTTTTTTTTTTTTTTTCGAAGAAATACTGCAACTTA |  |  |
|  | Spike 8 | Forward | TAATACGACTCACTATAGGGAAGGTCTGCTGGCGACCGG |  | 2000 |
|  |  | Reverse | TTTTTTTTTTTTTTTTTTTTTTTTTTTTTTACAGCATATGTTCAGTACG |  |  |
| qPCR | Spike 2 | Forward | GCCGCCAGTGCAGAAATC |  | 62 |
|  |  | Reverse | ATCAAAGTGCGGCAATTAAGC |  |  |
|  |  | Probe | ACTGTTCGTCACGAAAT | 5’ FAM/3’ NFQ, MGB |  |
|  | Spike 3 | Forward | GGCTTCGCTGAAGGTGTGA |  | 58 |
|  |  | Reverse | CGATGCTTTCCAGCTGTACCA |  |  |
|  |  | Probe | ATGGGCCATCAGTTG | 5’ FAM/3’ NFQ, MGB |  |
|  | Spike 6 | Forward | CCAACATTCAGCAGCTGAATGA |  | 59 |
|  |  | Reverse | CAGGAACGCTACTCGTGGA |  |  |
|  |  | Probe | CCAGCGTGGCGCTTTG | 5’ FAM/3’ NFQ, MGB |  |
|  | Spike 8 | Forward | CCTCCGGTACTGACCATCTTG |  | 68 |
|  |  | Reverse | CGCTGCCGTAGGCAGTTTAC |  |  |
|  |  | Probe | TGCGGTCATTGGCTCA | 5’ FAM/3’ NFQ, MGB |  |

Supplementary Figure 1. Relative abundances of transcripts in bead-supported cDNA libraries obtained by qPCR. The copy number of gene RPS18 was set to 100% as a reference for calculating relative ratios of cDNA for other three genes (RPL13A, GUSB, and ATP5B). The ratios were obtained with samples after RT (blue column) and after exonulease-I treatment (red column). The error bars are independent amplification replicates.

Supplementary Figure 2. Average gene expression for single cells as well as cell pools including 10, 100 and 1000 cells respectively. The primer and beads number for one-tube RT is 4,000 primer molecules per beads and total 10^7^ beads. The cDNA molecular of four genes (EEF1G, B2M, TBP and SDHA) in RT products was determined by qPCR.

Supplementary Figure 3. Evaluation of amplification efficiency (a) and relative amplification bias (b) for spike 2, spike3, spike 6 and spike 8 with the proposed bead-based method. A set of spike-in transcripts (10 copies of spike 2, 50 copies of spike 3, 200 copies of spike 6 and 1000 copies of spike 8) was added into RT reaction solution containing transcriptomes from single cell. The cDNA copies of the four spike-in transcripts in the 2^nd^ PCR products were quantitatively analyzed by qPCR. The amplification factors for the four spike-in transcripts were obtained by averaging the ratios of the cDNA copies after the amplification (n=5) to the adding copies before the amplification. The geometric means were calculated to obtain the amplification bias (amplification bias: the ratio of an amplification factor to the geometric mean).

Supplementary Figure 4. Evaluation of relative amplification bias for cDNA of twelve genes (RPL4, RPS18, RPL13A, ESD, FOS_1, ICT1, GTSF1, PGK1, GAPDH, ALDOA, YWHAZ_2 and HMBS) with the proposed bead-based method.
